# Addressing Latent Tuberculosis: New Advances in Mimicking the Disease, Discovering Key Targets, and Designing Hit Compounds

**DOI:** 10.3390/ijms21228854

**Published:** 2020-11-23

**Authors:** André Campaniço, Shrika G. Harjivan, Digby F. Warner, Rui Moreira, Francisca Lopes

**Affiliations:** 1 Instituto de Investigação do Medicamento (iMed.ULisboa), Faculdade de Farmácia, Universidade de Lisboa, Av. Prof. Gama Pinto, 1649-003 Lisboa, Portugal; andrecampanico@campus.ul.pt (A.C.); sharjivan@ff.ulisboa.pt (S.G.H.); rmoreira@ff.ulisboa.pt (R.M.); 2 Institute of Infectious Disease and Molecular Medicine, University of Cape Town, Rondebosch 7701, South Africa; digby.warner@uct.ac.za; 3 Department of Pathology, SAMRC/NHLS/UCT Molecular Mycobacteriology Research Unit, University of Cape Town, Rondebosch 7701, South Africa; 4 Welcome Centre for Infectious Diseases Research in Africa, University of Cape Town, Rondebosch 7701, South Africa

**Keywords:** *M. tuberculosis*, latency, mimicking latency, drug discovery

## Abstract

Despite being discovered and isolated more than one hundred years ago, tuberculosis (TB) remains a global public health concern arch. Our inability to eradicate this bacillus is strongly related with the growing resistance, low compliance to current drugs, and the capacity of the bacteria to coexist in a state of asymptomatic latency. This last state can be sustained for years or even decades, waiting for a breach in the immune system to become active again. Furthermore, most current therapies are not efficacious against this state, failing to completely clear the infection. Over the years, a series of experimental methods have been developed to mimic the latent state, currently used in drug discovery, both in vitro and in vivo. Most of these methods focus in one specific latency inducing factor, with only a few taking into consideration the complexity of the granuloma and the genomic and proteomic consequences of each physiological factor. A series of targets specifically involved in latency have been studied over the years with promising scaffolds being discovered and explored. Taking in account that solving the latency problem is one of the keys to eradicate the disease, herein we compile current therapies and diagnosis techniques, methods to mimic latency and new targets and compounds in the pipeline of drug discovery.

## 1. Introduction

The bacillus *Mycobacterium tuberculosis* (*M.tb*), causative agent for tuberculosis, was first identified in 1882 by Robert Koch. Koch was also responsible for the first extraction of tuberculin, a combination of proteins that would later become one of the basis of tuberculosis diagnosis [1]. Nowadays, more than 130 years after its discovery, *M. tuberculosis* remains a hot research topic and TB stands as one of the top 10 causes of death worldwide [2]. According to the latest reports, in 2018, 10 million new cases emerged, with 1.5 million people dying from the disease. Furthermore, 66% of the new cases occurred in specific countries: India, China, Indonesia, the Philippines, Pakistan, Nigeria, Bangladesh, and South Africa [3]. Besides the increasing resistance of the bacillus towards current therapies [4], one of the reasons for the success of this pathogen is its ability to coexist within the host in an asymptomatic latent state [5]. The phagocytosis of the bacillus by alveolar macrophages and dendritic cells along with the formation of the granuloma creates an avascular, inflammatory, and necrotic environment, in which the bacteria faces low oxygen levels and oxidative stress [6,7]. As a strictly aerobic microorganism, any decrease in the available oxygen leads to a significant decrease in bacillus growth, until it ceases completely [8]. The bacillus then activates a series of pathways leading to protein stability and homeostatic regulation, with maintenance of a basal metabolic level. *M.tb* adjusts energy sources, reduces energy expenditure, preparing itself for the dormancy state [9,10]. When this nonreplication is reached, the disease enters a state of latent infection. This dormancy state can be maintained for months, years, or even decades. It is estimated that 5 to 10% of individuals with this subclinical infection may develop clinical manifestations 2 to 5 years after the initial infection [11,12,13].

The current antitubercular therapy is a combination of four antibiotics: isoniazid, rifampicin, ethambutol, and pyrazinamide. The treatment regimen consists of a 2-month initial treatment, followed by a 4- or a 7-month continuation therapy. The initial phase uses the four drugs, with different mechanisms of action, each performing an essential role. Isoniazid and rifampicin show high cure rates in short-course regimens. Pyrazinamide is active against the latent bacillus, showing a potent sterilizing activity. Ethambutol prevents the emergence of resistance against rifampicin when resistance to isoniazid is present. The most common continuation phase is the 4-month therapy and it generally includes only isoniazid and rifampicin [14,15]. This long-term therapy is essential for a complete eradication of *M.tb* and to prevent dormant bacilli from remaining in the host [16]. However, and despite all these efforts, a study performed in 2014 estimated the global burden of latent tuberculosis as 23%, approximately 1.7 billion people, with the regions South-East Asia, Western-Pacific, and Africa accounting for 80% of this value [17]. The End TB Strategy sets 2035 as the year in which the number of deaths caused by the disease would have dropped by 95%. Not only to increase monitoring and therapy compliance, to reach this goal, it is essential to understand and find permanent and highly efficient solutions for latent tuberculosis [18].

## 2. Latent Tuberculosis Diagnosis and Current Therapies

The most common therapy for the latent tuberculosis infection (LTBI) is a 6-months’ daily monotherapy with isoniazid [14,19]. This was initially compared with the placebo, showing a significant decrease in TB incidence [20]. The efficacy of this therapy was also compared with twelve- and thirty-six-months treatments, with no significant differences [14,21]. Another analysis, however, suggests that 9 months is the optimal period for the treatment of LTBI using isoniazid monotherapy [22]. Therefore, the recommended duration for this therapy is considered 6 to 9 months [19]. Isoniazid advantages rely on its considerable clinical experience and low cost, however, the suboptimal compliance of such a long treatment and its hepatotoxicity led to alternative therapies [23,24,25]. Despite being used to treat LTBI, isoniazid is not active against nonreplicant bacillus, being used only as a preventive drug and requiring long therapeutic periods to prevent the progression into the active disease [26,27]. Furthermore, isoniazid has been shown to induce its own resistance when used in nonreplicant *M.tb*. After 1-week incubation in liquid media at concentrations of 0.1 and 0.4 µg/mL, subpopulations of bacilli were no longer susceptible to isoniazid. A similar experiment was performed with rifampicin and induction of resistance was not observed [28].

The development of techniques that mimic latency allowed insights on more efficacious drugs and shorter therapies. Overall, the most active drugs against dormant *M.tb* were the rifamycins (rifampicin and rifapentine), followed by pyrazinamide [29]. Three new treatment regimens are used as shorter and equally efficacious therapeutic alternatives for LTBI: 3-, 4-months’ daily rifampicin monotherapy; 3-, 4-months’ daily rifampicin plus isoniazid; 3-months’ weekly rifapentine plus isoniazid [14,19]. The use of rifampicin has been associated with lower hepatotoxicity and higher completion rates when compared with isoniazid in 6-months’ monotherapy [30,31]. Rifapentine displays longer half-life and increased potency than rifampicin, allowing weekly treatments with similar efficacy. Despite the efficacy of pyrazinamide against dormant bacilli, this drug is not in any of the recommended therapies due to its high hepatotoxicity [32,33].

The recommended methods to diagnose LTBI are the tuberculin skin test (TST) and the interferon-γ release assay (IGRA) [34]. For nearly one hundred years, TST was the only test available. It involves the injection of the tuberculin purified protein derivative (PPD) intradermally on the forearm and this area is then examined 48 to 72 h later. A delayed-type hypersensitivity reaction is observed in all individuals that were already sensitized for the antigens in PPD [35]. The major limitation of this test is the false-positive obtained from people who have been administered with the Bacillus Calmette–Guérin (BCG) vaccine [36]. IGRA is an in vitro test that measures the interferon-γ produced by lymphocytes, after incubation with the antigens early secretory antigen target-6 (ESAT-6) and culture filtrate protein-10 (CFP-10). The main advantage of ESAT-6 and CFP-10 is their absence from the BCG vaccine, overcoming the main problem of TST [35,37]. However, false positives may occur from infection with other mycobacteria species [37,38]. The sensitivity of both tests can be compromised in HIV-infected individuals with low CD4 T-cells count and patients on immunosuppressive drug therapy [39,40]. Furthermore, and since they are both indirect methods to detect an *M.tb* infection, it is not possible to distinguish individuals who are latently infected from those who have cleared the infection but still have a persistent anti-mycobacterial immune response [39]. This major drawback can favor the administration of anti-tubercular therapy on healthy individuals, increasing the disease-related costs and the possibility of unnecessary adverse effects [41].

## 3. Methods to Mimic Latency

In order to reach a nonreplicative state of *M.tb* for experimental purposes, it is essential to understand the conditions within the granuloma and mimic them as faithfully as possible [42]. The granuloma is characterized by a variety of stress conditions, being the most studied hypoxia, nutrient deprivation and limited carbon sources, and high concentration of nitric oxide [43,44,45]. The combination of all these factors would ideally replicate the phenotype found in the latent interaction between pathogen and host. However, and despite a few cases where several of these conditions are met, the majority of in vitro models is based on only one of the factors involved in latency [42].

### 3.1. Hypoxia

Hypoxia is the best characterized factor that triggers the latency state [46]. The first and most famous model was developed by Lawrence Wayne, upon the observation of arrested growth in the event of discontinued aeration of an *M.tb* culture [47]. This state, similar to latency, but with subtle differences, was then named nonreplicating persistence (NRP) [8,48]. The Wayne model, introduced in 1996, aims to simulate the depletion of oxygen within the granuloma and reach that NRP state. The *M.tb* growth occurs in a sealed container, with a controlled air ratio. As the available oxygen is consumed, the organisms slowly shift to anaerobic conditions. Two different NRP stages can be observed. Stage I is attained when the oxygen saturation achieves 1% and, even though the bacilli are not replicating or synthesizing DNA, there is still high production of ATP and some mechanisms of DNA repair remain functional [48,49,50]. When the oxygen saturation reaches 0.06%, the culture enters Stage II, reaching full anaerobic conditions. Even though NRP Stage II is often described as simply NRP, it is not possible for an organism to survive by being placed directly in Stage II. A gradual decrease in oxygen levels is essential [8,48,49,50]. When screened under this model, *M.tb* is resistant to isoniazid but susceptible to metronidazole, a drug with no inhibitory effect in aerobic conditions [51,52]. This bactericidal effect was also observed in non-human primate and rabbit models but was not reflected in mice, putting into question the activity of compounds identified using the latter model [7,53,54,55]. The reason for this difference lies on the methodologies itself. Being based only on hypoxia, the Wayne model generates bacilli with physiological and metabolic profiles different from other methods used for in vivo studies [53]. Over the years, adaptations of the Wayne model have been developed, in order to facilitate the screening of new drugs [42].

The hypoxic resazurin reduction assay (HyRRA) is a conjugation of the Wayne model with the resazurin microtiter assay (REMA) [56]. This colorimetric assay uses alamar blue for the identification of the minimum inhibitory concentration (MIC) of compounds against the NRP *M.tb*. After cultures reach the hypoxic and NRP state, the drugs are added and the system is incubated for 96 h. The resulting cultures are then dispensed into microtiter plates and the alamar blue is added to the wells. The viability of these cells is evaluated, and the MIC is determined by the colorimetric result [57]. Despite being a promising conjugation of methods, it may fail to identify potential compounds since 96 h may not be enough for some drugs to display activity [42].

Another adaptation of the Wayne model is the low oxygen recovery assay (LORA) [58,59]. This assay measures the metabolic activity levels of the culture by taking advantage of a luciferase reporter (luxAB gene) [60,61]. When entering the NRP state, the luminescence of the culture decreases, allowing the method to be used in high-throughput screening (HTS). The hypoxia is reached as previously described and a chemostat can be used to accurately control the oxygen levels [62].

In 2008, Yeware and Sarkar developed another method derived from the Wayne model, using red fluorescent protein (RFP) to measure the culture growth [63]. RFP appears to give a stronger and more stable signal in dormant mycobacteria than other reporter genes, such as green fluorescent protein (GFP) and the luciferase reporter (used in LORA) [62,64,65]. Furthermore, hypoxia was achieved by adding a layer of paraffin oil, impermeable to oxygen. The compounds for the HTS were inserted directly through the paraffin layer. Contrary to other hypoxic models, metronidazole showed no activity against the NRP bacilli using this method [63].

### 3.2. Nutrient Deprivation

Nutrient deprivation was early identified as a way to reach the NRP state. In 1933, Loebel et al. observed that it was possible to transfer a *M.tb* culture from a rich media to PBS and leave it for many years in solution [44]. The bacilli remained viable, but their respiration levels were slowly decreased as the culture entered a stationary state [66]. When reintroduced to a rich media, the bacteria resumed normal growth [44]. These findings were the basis of the first NRP state model based on nutrient deprivation, the nutrient deprivation model. Developed in 2002 by Betts et al., this method predicts the growth of mycobacteria in a rich media for 7 days, followed by centrifugation and resuspension in PBS. The viability of the culture was determined by colony-forming unit (CFU) counts in specific timepoints. Despite being kept in a sealed container, like in the Wayne model, methylene blue analysis reflected the presence of oxygen in the media [67]. Being deprived from ‘foodstuff’, the bacteria reduced their respiration rates and entered the NRP state, regardless of oxygen availability [66,67]. When screened against antibiotics, the new culture gained resistance to isoniazid without acquiring susceptibility to metronidazole [67]. This difference to hypoxia can be explained by the different transcriptomes derived from the two different conditions [68].

In 2004, Hampshire et al. developed a method of progressive nutrient depletion, measured by a chemostat. Apart from reporting the carbon sources, the device allowed a stable control over pH, temperature, and oxygen levels. After a quick depletion in glucose and a gradual depletion in glycerol, the culture reached a stationary state by day 15. The exhaustion of resources led to a progressive drop in viability until day 78. On day 78, a small population of fully adapted bacilli restarted to grow, reaching 4-fold of the previous measurement by day 111. Analysis of these 3 phases showed notorious differences, with the second phase, from day 15 to 78, matching the expected transcriptomic alterations for an NRP state [69].

### 3.3. Nitric Oxide

Even though hypoxia and nutrient starvation are the most studied environmental conditions of the granuloma, they are not the only parameters that can induce the NRP state [42]. Nitric oxide (NO) was identified as capable of inducting the expression of *M.tb* dormancy regulon genes at nanomolar concentrations, causing the bacillus to enter dormancy in vitro [70,71,72]. By inhibiting mycobacterial respiration and halting replication, NO generates an NRP state similar to the Wayne model and hypoxic conditions [71]. No functional model has yet been developed based on nitric oxide, however it would be expected a phenotype comparable to the one observed for hypoxia [42].

### 3.4. Streptomycin-Dependent Model

In 1955, Hashimoto et al. isolated the *M.tb* strain 18 b from a patient with tuberculosis. This strain was discovered to be not only resistant to streptomycin, but also streptomycin-growth dependent. Despite these findings, this strain remains viable in streptomycin-free media for several weeks, without multiplying, resuming growth upon the adding of the antibiotic [73,74]. Later, in 2010, Sala et al. exploited the *M.tb* strain 18 b for its potential as an NRP model. They observed that the growth rate was directly correlated with the concentration of streptomycin in the media. Looking into the phenotype, this strain entered the NRP state after 10 days without antibiotic, maintaining it until streptomycin was reinstated in the culture [75]. When analyzed for drug susceptibility, isoniazid showed no activity but rifampicin displayed an increased efficacy. New compounds in clinical trials were also tested with positive results. For HTS purposes, this strain was screened using the REMA technique already described [75,76]. This model has been used in drug discovery, not only allied with REMA, but also with the luciferase assay [77,78].

### 3.5. Multiple Stress Models

Despite the success of single factor stress models, more complex and multifactorial methods replicate more accurately the environment within the granuloma. In 2009, Deb et al. reached the NRP state of *M.tb* with a low-nutrient and acidic medium (pH 5), using an atmosphere with 10% CO_2_ and 5% O_2_ for 18 days. By screening the colony at day 18, the bacilli were resistant to rifampicin by 310-fold and to isoniazid by 2800-fold, when compared to day 0 [79]. When the results are compared to the Wayne model, the bacteria show a much higher resistance to rifampicin in this multi-stress method [8,79]. Another multi-stress model was purposed by Gold et al. in 2015. To reach the dormant state, the culture was exposed to a mildly acidic environment (pH 5), a flux of nitric oxide, and other reactive nitrogen intermediates, hypoxia with 1% oxygen levels and fatty acids as the carbon source. The assay was divided into two phases: a first phase with nonreplicant bacilli exposed to the drugs for 3 to 7 days; a second phase of outgrowth, that would facilitate the reading of viable cells. In order to prevent false positives in compounds that were only active in the outgrowth phase, a second assay was performed in parallel with replicating *M.tb* and the results were compared [80]. Despite these models being the most complex and granuloma-like available, still relevant host factors are not being taken into consideration. Examples of these conditions are deprivation of iron, magnesium, amino acids, and vitamins; copper intoxication; exposure to reactive oxygen intermediates and carbon monoxide [81,82,83,84,85,86,87,88,89,90].

### 3.6. In Vivo Models

Several animal models have been used to replicate the latency of *M.tb* [91]. Even though mice would be the most common choice due to its convenience, low-cost, availability, and technical benefits, the bacterial burden observed in these animals is a lot higher, with crucial differences in the structure of the granuloma [92,93]. Other models such as guinea pigs, rabbits, and non-human primates have also been studied, with advantageous similarities to humans [94]. Zebrafish and medaka models have been developed, recurring to *Mycobacterium marinum*. Analyzing these last two methods, zebrafish appears to be more useful when representing the active infection, while medaka is more representative of the latency and chronic tuberculosis [95,96,97]. Since our main goal is to analyze latency models that can be used in drug development for *M.tb* and as zebrafish and medaka models use *M. marinum*, these will not be further explored in this paper.

The first model for in vivo latent tuberculosis was developed at Cornel University, by McCune and McDermott in 1966. For this method, the mice were infected with a high burden of bacilli for a long period and then treated with isoniazid and pyrazinamide until the levels of mycobacteria were undetectable. Once this state was achieved, they observed that the remaining ‘persisters’ could cause relapses by cessation of therapy or by injection of corticosteroids [98,99]. More accurately, the Cornel model is described as a model of *M.tb* persistence rather than latency, since the undetectable state is achieved by drug treatment and not by defense mechanisms of the host immune system [91,98,99]. However, this low bacterial burden is still similar to latent tuberculosis in humans and this method has been used in drug discovery [100]. Another disadvantage of this method is the premature death of several mice due to the high bacterial burden imposed [91,98,99]. A different approach to obtain an NRP state within the mice was demonstrated by directly applying the Wayne model in vivo. The animals are infected with NRP bacilli and it was observed that this state is maintained for slightly less than 10 days. This variant of the Wayne model was used to measure the impact of vaccination in the reactivation of *M.tb*, but could be applied to antimycobacterial drug discovery following the same rational [8,101]. The streptomycin-dependent model has also been applied in vivo. The mice were infected with the streptomycin-dependent mutant strain and injected with streptomycin for 3 weeks. After this period, the drug administration was interrupted, and the bacilli entered the NRP state [78]. Despite all these advances, none of these methods was able to replicate the immune response that generated the latency state. An immune-based murine model was firstly developed in 2004. The mice were immunized using a recombinant BCG strain and infected with the bacteria 6 weeks later. The immunization overcame the problem of the premature death of mice and allowed a state of bacterial burden very similar to latent tuberculosis in humans [102,103]. After its development, the method was enhanced in 2014, by using C3Heb/FeJ mice, a mutant variation that allows the development of necrotic lung granulomas and reactivation with TNF-α [104]. Finally, a novel hypoxic granuloma method was also proposed, with the encapsulation of the bacteria in semi-diffusible hollow fibers and subsequent subcutaneous implantation. Once encapsulated in these fibers, *M.tb* upregulates the expression of several genes also expressed in the mouse model of chronic tuberculosis. Furthermore, genes involved in metabolism and DNA replication were downregulated, matching the expected physiological profile of persistent bacilli. The main drawback of this method is the site of infection, different from the physiological environment of the lungs [105].

Guinea pigs display a granuloma similar to humans, with traits of necrosis and hypoxia [7,91]. However, they still show some similarities with mice, exhibiting high bacterial burden infections and premature death, with low tendency to latency and chronicity [91]. A similar rational to Cornell model was developed, with guinea pigs being subcutaneously infected with *M.tb* and receiving drug treatment until the bacterial load was undetected [106]. In this particular model, the authors stated that there is no significant difference between subcutaneous and aerosol administration when it comes to demonstrating the efficacy of vaccines, which was their goal [107]. The streptomycin-dependent model has also been used in guinea pigs in a procedure identical to mice [108].

Rabbits are considered the most resistant animal model to *M.tb*, despite developing granulomas similar to humans and guinea pigs [7,109]. In 2008, Manabe et al. showed that the aerosol infection of rabbits with *M.tb* naturally evolved from an active infection state to latency in 72% of the hosts, with undetectable bacterial levels but reactivation with immunosuppressant therapy [109]. Later, in 2012, a similar study was conducted. Rabbit infection with *M.tb* led to an exponential bacterial growth and active infection for 4 weeks, after which the bacterial load gradually decreased. By week 20, all subjects showed undetectable levels. After 4 weeks of immunosuppressant treatment, *M.tb* resumed an active growth state, showing that the apparent bacterial clearance was not a result of sterilization but of latency and chronic infection. Rabbits can then be characterized as natural and spontaneous models for latent tuberculosis [110,111].

The efficacy of vaccines and drugs has been tested in both rhesus and cynomolgus macaques, however, rhesus macaques are more susceptible to the infection than cynomolgus, being more appropriate for the study of the active disease [112]. Cynomolgus macaques are the model that better represents latency and the chronic tuberculosis [113]. Studies have shown that a low-bacterial dose infection of cynomolgus macaques may lead to active or latent tuberculosis. The chronic bacilli were naturally reactivated in a low percentage of hosts [114,115,116,117,118,119]. The non-human primate has been described as the model that most accurately reproduces the traits of the latent *M.tb* infection in humans [120].

## 4. Targeting Nonreplicating *M.tb*

The growing understanding of the latent phase of *M.tb* and its implications in the reactivation of the disease led to the search for compounds that can block latent form activation or target active enzymes during the dormant phase. The combination of bioinformatic prediction and genomic studies allowed the identification of several potential targets, crucial for survival during this phase [121,122]. Furthermore, the screening of new compounds against dormant bacilli increased in the past few years, contributing to the identification of new potent scaffolds with sterilizing effects on *M.tb*.

### 4.1. Alanine Dehydrogenase and Isocitrate Lyase

**Figure 1:**
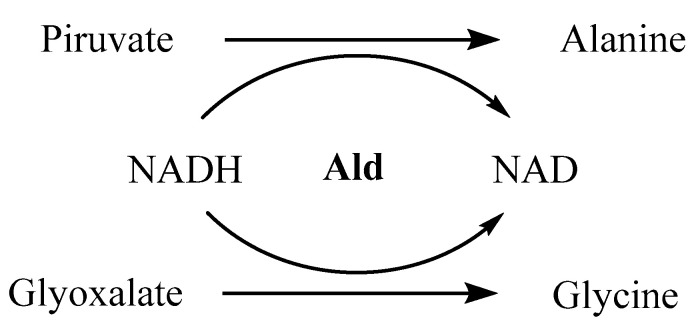
Catalytic function of alanine dehydrogenase.

When entering the latent state, *M.tb* adapts to hypoxia by remodeling its metabolic routes for anaerobic pathways, with alternative electron acceptors being activated [123,124,125]. One of the enzymes upregulated in this process is the alanine dehydrogenase (Ald), whose function is the conversion of pyruvate into alanine and glyoxalate into glycine, with oxidation of NADH to NAD (Figure 1). Besides playing a critical role in maintaining the redox balance NADH/NAD, alanine is then used as a nitrogen source for new electron acceptors [126]. Glyoxalate and pyruvate are supplied by the glyoxalate and methylcitrate cycles, respectively, with the two isoforms of isocitrate lyase (Icl1 and Icl2) being responsible for the catalysis of its formation [127]. Expression of both enzymes has been analyzed under hypoxia, using the Wayne model [8]. It was observed that the levels of Ald were upregulated 5-fold in NRP-1 and 16-fold in NRP-2, while Icl1 was increased 39-fold in NRP-1 and downregulated in NRP-2 and Icl2 was similar to Ald. All these values are calculated in comparison with its expression in an aerobic state. Ald and Icl have therefore been considered as latency specific targets over the past few years [128,129,130].

**Figure 2:**
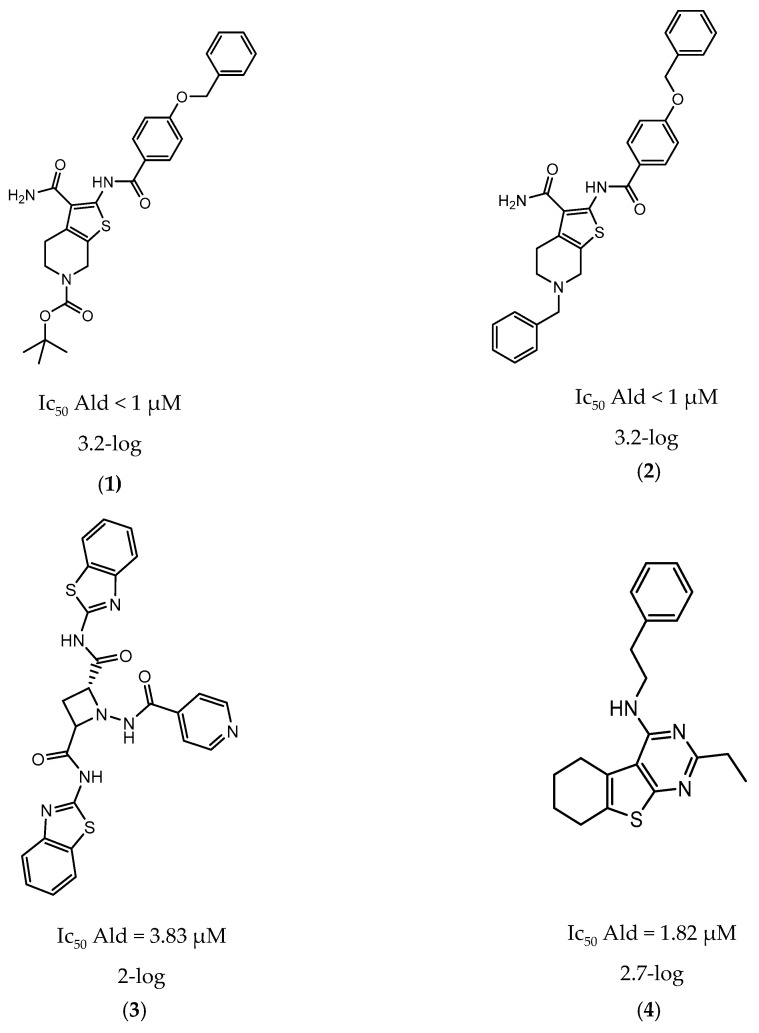
Selected Alanine Dehydrogenase (Ald) inhibitors (**1**, **2**, **3**, **4**), with respective Ald IC_50_ and bacterial reduction values.

In 2014 and 2015, Saxena et al. successfully identified several Ald inhibitors using molecular docking studies. Some of these compounds were synthesized and tested against both active and dormant bacilli, showing modest to potent activities in active bacteria and effectively inhibiting dormant cells. This last screening was performed using the Betts model [67] and two compounds (**1** and **2**, Figure 2) were identified as causing a bacterial reduction of 3.2-log, showing also an IC_50_ for the *M.tb* Ald below 1 µM [131,132]. In 2016, the same group synthesized another library of inhibitors, with the most promising compound (**3**, Figure 2) displaying a 2-log bacterial reduction and an IC_50_ of 3.83 µM [133]. Following a whole-cell screen reported by GSK, Samala et al. were also able to identify Ald inhibitors, with one compound (**4**, Figure 2) providing a bacterial reduction of 2.7-log using the Betts method [67]. The interaction with the enzyme was confirmed using differential scanning fluorimetry, with the referred compound showing a thermal shift of 1.4 °C and an IC_50_ of 1.82 µM. Despite the activity against dormant strains, the MIC for active bacilli was weak, with values of 37.04 µM, suggesting that the scaffold still needs further optimization [134].

**Figure 3:**
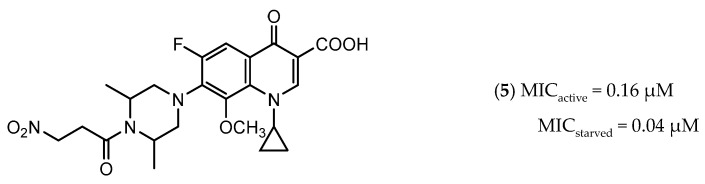
Isocitrate Lyase (Icl) inhibitor with described Minimum Inhibitory Concentration (MIC) against active and starved bacilli.

Icl inhibitors were reported by Sriram et al. in 2011, when a full library of 3-nitroproprionamides was synthesized [135]. 3-Nitroproprionate was already a known inhibitor of Icl [136]. The newly synthesized compounds were shown to inhibit the enzyme, with IC_50_ below 1 µM, and displayed high selectivity as no cytotoxicity against mammalian cells was observed. They were screened against bacilli in the active and dormant phase (achieved through starvation) with promising MIC. The most potent compound (**5**, Figure 3) exhibited an MIC of 0.16 µM against active bacilli and 0.04 µM against starved *M.tb* [135]. Other Icl inhibitors have been successfully identified, however none of them were tested against dormant *M.tb* forms [137,138].

### 4.2. Sulfur-Mediated Redox Homeostasis

After phagocytosis, the macrophages kill the bacteria engulfed in phagolysosomes using reactive oxygen and nitrogen intermediates (ROI and RNI) [139]. Therefore, the intracellular survival of any phagocytosed pathogen is dependent on its ability to use redox defense mechanisms. In the particular case of *M.tb*, this homeostasis is assured by mycothiol, an analog of glutathione [140]. Possessing a cysteine-derived sulfhydryl group, its availability and biosynthesis is directly related to the biosynthesis of *L*-cysteine and the sulfur metabolic pathways [141]. Bearing its crucial role in intracellular survival, these enzymes were observed to be upregulated in several dormancy models, with potential to be used as future drug targets [49,67,142]. Two of these enzymes already have inhibitors described in literature, those being the cysteine synthase CysM and the adenosine-5′-phosphosulfate reductase (APSR) [143,144].

**Figure 4:**
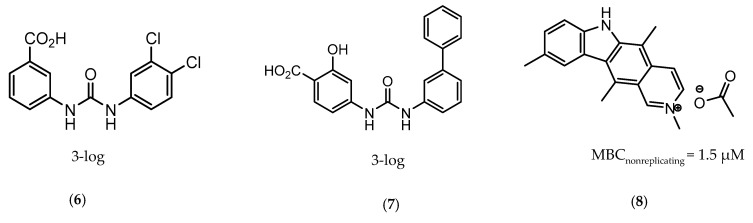
Selected Cysteine Synthase CysM (**6**, **7**) and Adenosine-5′-phosphosulfate Reductase (APSR) (**8**) inhibitors, with respective bacterial count and Minimum Bactericidal Concentration (MBC) against non-replicating bacilli values.

CysM is one of the three pyridoxal-phosphate (PLP)-dependent cysteine synthase present in *M.tb*. This enzyme catalyzes the synthesis of cysteine using O-phosphoserine and CysO, a sulfur delivery protein [145]. Although two other enzymes can be alternatives towards the biosynthesis of cysteine, the resistance and stability of the intermediate in the catalytic reaction of CysM favors this route under oxidative stress. Therefore, the CysM catalysis is the dominant pathway for the synthesis of cysteine in dormant bacilli [146]. Targeting CysM, a family of inhibitors was developed [143,147]. Since the genomic expression of this enzyme is more prevalent in the dormant state, [67,142] as expected, when tested against active *M.tb*, these compounds only showed modest activities. However, its promising K_d_ values (ranging between 0.32 and 8 µM) matched good screening results when they were tested against starved bacilli, with two compounds (**6** and **7**, Figure 4) showing a 3-log reduction in bacterial count [143].

The enzymes involved in the sulfate assimilation pathway allow the targeting of a different part of the sulfur mycobacterial metabolism, leading to reduced sulfur levels for the biosynthesis of cysteine and other metabolites [148,149,150]. Furthermore, the expression of these proteins has been identified as upregulated in the dormant state and in situations of oxidative stress [67,69]. Adenosine-5′-phosphosulfate reductase (APSR) is a critical step of this route, reducing the sulfate in adenosine-5′-phosphosulfate (APS) to sulfite and adenosine-5′-monophosphate (AMP) [151,152]. Besides its connections with oxidative stress and mycobacterial survival, this enzyme does not have a homologue in humans, turning it into a potentially attractive and selective therapeutic target [144]. Over the years, inhibitors of APSR have been reported, either discovered by HTS or designed as analogs of its substrate, APS [153,154,155]. However, only in 2016, a series of inhibitors derived from HTS were for the first time screened against nonreplicating *M.tb*, confirming the potential of this target for dormant bacilli. From this family of inhibitors, two compounds showed minimum bactericidal concentration (MBC) against nonreplicating bacilli of 3 µM and the most promising compound (**8**, Figure 4) displayed an MBC against nonreplicating bacilli of 1.5 µM. The most potent inhibitors showed no cytotoxicity against VERO monkey kidney cell lines, validating the specificity of this target [144].

### 4.3. DevR/DevS/DosT System

Oxygen levels in cells are detected through more or less complex biochemical sensors, depending on the organism [156]. In the case of *M.tb*, its presence or absence is determined by DevS and DosT, two oxygen heme-based sensors, which function like a two-component system along with DevR, a response regulator [157]. By sensing variations in the levels of oxygen in the surrounding media, these promote a series of metabolic changes in the bacillus, initiating the process of latency. DevS and DosT work as receptors that sense the presence of oxygen and regulate their histidine kinase domain accordingly [158]. DosT, the sensor with the lowest oxygen affinity, is the first to be activated in a situation of hypoxia, followed by DevS, in even lower levels of oxygen [159]. These two receptors are then able to phosphorylate DevR, which leads to the upregulation of several genes and several phenotypical changes [160]. The crucial role of this system on initiating the dormancy state supports its importance in the virulence of the pathogen, turning it into a promising potential target for drug discovery [161].

**Figure 5:**
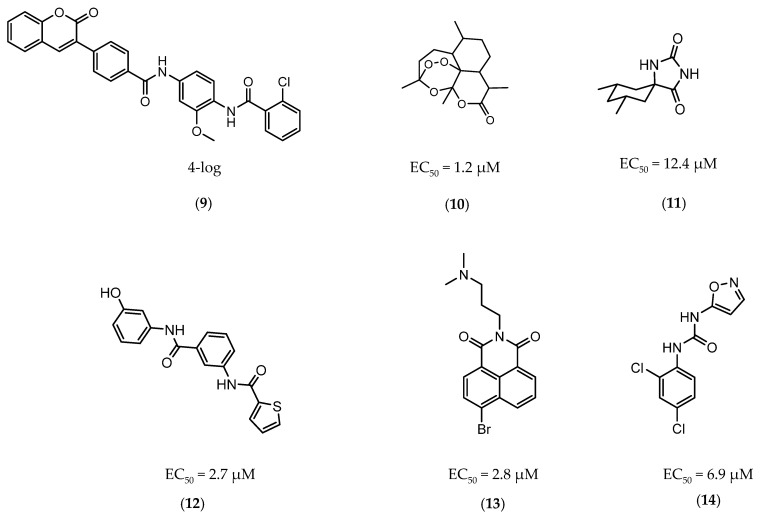
Selected DevR/DevS/DosT system inhibitors (**9**, **10**, **11**, **12**, **13**, **14**), with referred bacterial count and EC_50_ values.

The first DevR inhibitor was discovered in 2009 from a library of 2.5 million compounds, using an in silico pharmacophore-based screening. Compound **9** (Figure 5) was identified as inhibiting the binding of DevR to DNA, displaying activity against hypoxic cultures with a 4-log reduction in bacterial count. However, to achieve these results, a high concentration of this compound was necessary (131 µg/mL) and no inhibition was observed in aerobic or nutrient starved bacilli [162]. More recently, Zheng et al. performed an HTS to identify inhibitors of this system. Five compounds with five different scaffolds fitted in this profile, among them, the known antimalarial drug artemisinin (10, Figure 5) and compounds **11**–**14** (Figure 5). Although all compounds showed EC_50_ against a dosR-dependent mutant GFP reporter between 1.2 and 12.7 µM, none was able to efficiently inhibit active *M.tb* growth. Further studies, revealed that compound **10** downregulated 85 DevR-dependent genes (more than two thirds of all the genes regulated by this regulon) and 157 genes independent from this system, revealing lack of selectivity for the desired target. Compounds **11** and 12 displayed higher specificity, with 48 out of 55 and 76 out of 90, respectively, of the downregulated genes belonging to the DevR-regulated profile. These three compounds were also tested in a combined screening with isoniazid, with an increased sensitization to this drug being observed. The mechanism of action was also explored. While compound **10** led to the alkylation of the heme group of DevS and DosT, compounds **11** and **12** prevented the autophosphorylation of the sensors [163]. Later, compounds **13** and **14** were also further studied, with compound **14** displaying a higher specificity for this system and compound **13** inhibiting a significant portion of these genes but revealing off-target inhibition [164]. The impact of these compounds in survival during NRP state was accessed using a hypoxic model [165]. Compound **13** did not show any impact on survival, suggesting that the portion of genes it inhibits is not essential in the NRP state. On the other hand, compound **14** displayed a 50% survival rate when compared to the positive control. Their mechanism of action was also studied, with compound **13** preventing the binding of DevR to DNA and compound **14** binding to the heme group of DevS, disturbing its electronic spectrum [164].

### 4.4. Lysine ε-Aminotransferase

Lysine ε-aminotransferase (LAT) is a pyridoxal-5′-phosphate (PLP)-dependent aminotransferase, responsible for the catalysis of the conversion of lysine and *α*-keto glutaric acid into piperidine-6-carboxylic acid and glutamate, respectively [166,167]. The deletion of the lat gene revealed its important role in maintaining the amino acid pool and, subsequently, the levels of guanosine pentaphosphate, essential in inhibiting the RNA synthesis and promoting survival in a latent state [168]. The Betts model identified LAT as upregulated in dormancy about 41.86 times, turning it into an interesting target against latent *M.tb* [67].

**Figure 6:**
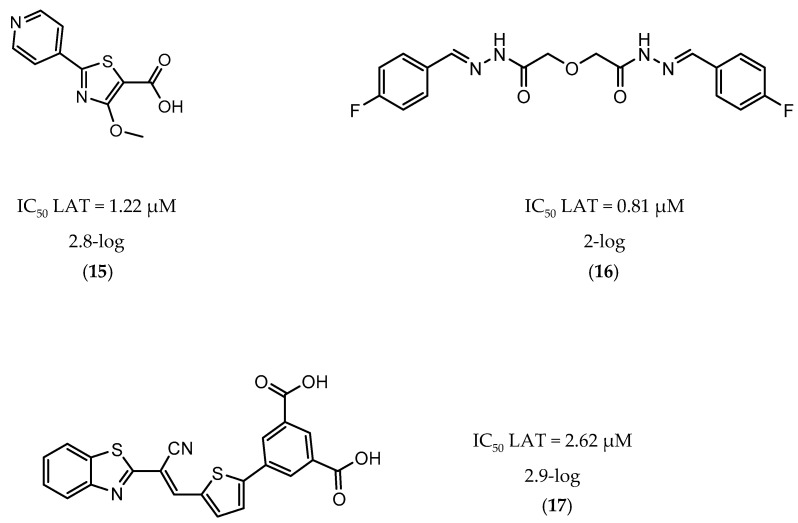
Selected Lysine ε-aminotransferase (LAT) inhibitors (**15**, **16**, **17**), with respective LAT IC_50_ and bacterial reduction values.

A few inhibitors have been described by the group of Dharmarajan et al. Their scaffolds were identified through a combination of structure-based and ligand-based methods with the crystallographic structure of LAT. Selected scaffolds were further explored and optimized, generating compounds with potent IC_50_ for the enzyme and significant inhibition against nutrient starved bacilli. The most promising compounds were compound **15** (Figure 6), with a 2.8-log bacterial reduction and an IC_50_ of 1.22 µM; compound **16** (Figure 6), with a bacterial reduction of over 2-log and an IC_50_ of 0.81 µM; and compound **17** (Figure 6), with a 2.9-log reduction in bacterial count and IC_50_ of 2.62 µM [169,170,171].

### 4.5. Other Targets

Besides targets specifically related with the progression and maintenance of dormancy, over the past few years, compounds targeting well-known enzymes essential for *M.tb* survival have also been tested against nonreplicating bacilli [2]. The enoyl-acyl carrier protein reductase (InhA), decaprenyl-phosphoryl-ribose 2′-epimerase (DprE1), mycocyclosin synthase (Cyp121), and extracellular zinc metalloprotease 1 (Zmp1) are examples of successful screenings. 

**Figure 7:**
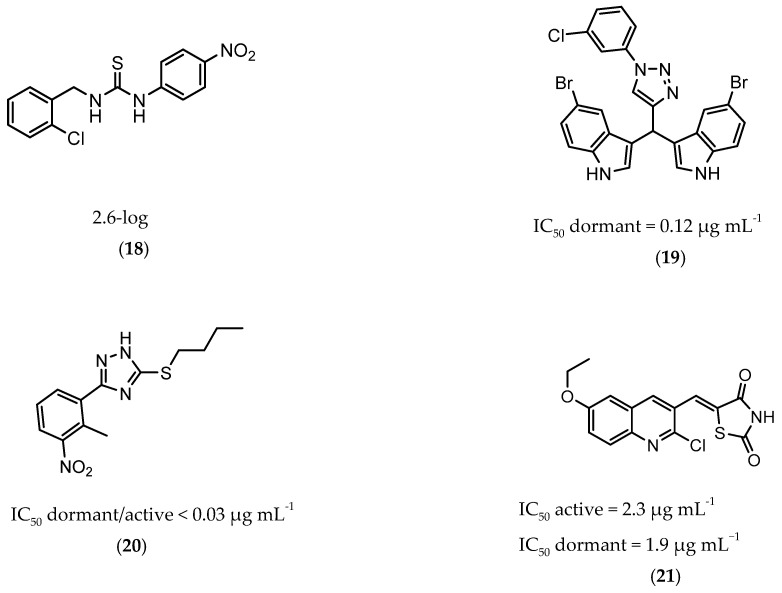
Selected compounds active against dormant *M.tb* (**18**, **19**, **20**, **21**), with referred bacterial count or IC_50_ for active/dormant bacilli values.

InhA is part of the fatty acid synthase (FAS)-II system, responsible for the catalysis of the synthesis of fatty acids using Acetyl-CoA [172]. InhA is the target of the first-line drug isoniazid that requires activation from a catalase-peroxidase enzyme, KatG, to act on its target [173,174]. Even though isoniazid is reported as inactive against dormant tuberculosis, [26] Doğan et al. screened their new family of thiourea-based derivatives against nutrient starved mycobacteria. The most promising compound (**18**, Figure 7) displayed a 2.6-log reduction in bacterial count. Activity against active bacilli and *M.tb* in infected macrophages was also confirmed [175].

DprE1 is one of the enzymes responsible for the cell wall biosynthesis, catalyzing the formation of decaprenyl-phosphoarabinose from decaprenyl-phosphoribose [176]. This target has been extensively explored as several scaffolds have been discovered and optimized for its inhibition [177,178,179,180]. In 2018, a family of triazole-diindolylmethane derivatives was designed from a molecular hybridization approach, by combining different pharmacophores. All compounds were tested against an attenuated strain (*M.tb* H37Ra), in both active and dormant state. Compound **19** (Figure 7) proved to be the most potent, with an IC_50_ of 0.12 µg mL^−1^ for nonreplicating bacilli [181].

CYP121 is an enzyme from the family cytochrome P450 that catalyzes the formation of mycocyclosin from cyclodityrosine, by coupling two tyrosine residues and generating a carbon-carbon bond [182,183]. This enzyme is essential for the survival of *M.tb* and cannot be found in any other microorganism or human cell, presenting itself as a specific target for the bacillus [184]. A series of 3-aryl-5-(alkyl-thio)-1H-1,2,4-triazoles derivatives was developed and was found to inhibit CYP121. These compounds were tested against H37Ra, with the most active compound (**20**, Figure 7) showing IC_50_ of <0.03 µg mL^−1^ against both dormant and active bacilli [185].

Zmp1 is an enzyme responsible for preventing the fusion between the phagosome and the lysosome within the macrophage [186,187]. This zinc-peptidase is crucial for the intracellular survival of the bacillus as it averts the phagosome maturation, playing a key role in its pathogenicity [188]. Conjugates of rhodanine and quinoline were synthesized and identified as Zmp1 inhibitors. All compounds were screened against the H37Ra strain in both active and dormant forms. The most promising compound (**21**) is depicted in Figure 7, showing IC_50_ of 2.3 µg mL^−1^ for active *M.tb* and 1.9 µg mL^−1^ for dormant *M.tb* [189].

All compounds referred in the previous section are depicted in Table 1.

## 5. Conclusions

The End TB Strategy intends to diminish the deaths from tuberculosis by 95% and sets 2035 as the year for this goal to become a reality [18]. Tuberculosis is nowadays one of the top ten causes of death worldwide and is responsible for one in every three deaths in people infected with HIV [3]. Thus, eradicating this disease in fifteen years is now a major challenge for the scientific community. Resistance is growing on first-line drugs and current drugs require long therapeutic periods. The subsequent low compliance that results from this last factor fails to completely cure the patient and, in most cases, leads to latent tuberculosis that can eventually turn active and restart the problem. Furthermore, the first-line treatment for the latent illness requires once again long treatments, is not effective in dormant bacilli and has been proved to induce its own resistance when used in monotherapy [28].

The goal set up by the WHO of eradicating TB by 2035 can still be accomplished but, this endeavor requires therapies to be highly monitored and more effective drugs to reach the market. More specifically, drugs that efficaciously cure the disease and are active against latent *M.tb* need to move forward in the pipeline and generate shorter and more effective treatments. More complex and accurate methods to mimic the latent state are needed. Most of the techniques used nowadays only take one latency inducing factor into consideration and it is common that the same drug exhibits contradictory results in different methods. The development of the latent state is complex, not fully understood and involves a series of factors that differently impact genetic modulation. A faithful reproduction will not be easy to accomplish. Most techniques currently used for drug screening against the latent state have not been updated for years and an effort needs to be made by the scientific community in order to generate more accurate mimicking methods that will increase the scientific impact of new compounds.

In order to fully eradicate the disease, this latency needs to be completely understood, specifically targeted, and successfully prevented. Tuberculosis must be addressed as a three-way problem: active disease, resistance to current drugs, and latency. Despite being equally important, research on compounds that are effective in the latent state has not been treated as a priority and, as long as fighting this nonreplicating bacilli is not fully embraced, *M.tb* will remain a problem to be solved.

## Figures and Tables

**Table 1:** Compounds active against dormant *M. tuberculosis*.

Compound	Structure	Target	Activity	Bacterial Reduction in Dormant Bacilli
**1**	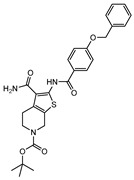	Ald	IC_50_ Ald < 1 µM [132]	3.2-log [132]
**2**	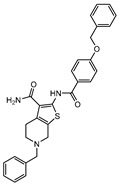	Ald	IC_50_ Ald < 1 µM [132]	3.2-log [132]
**3**	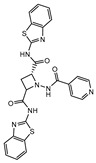	Ald	IC_50_ Ald = 3.83 µM [133]	2-log [133]
**4**	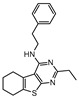	Ald	IC_50_ Ald = 1.82 µM [134]	2.7-log [134]
**5**	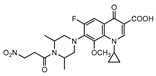	ICl	MIC_active_ = 0.16 µM [135] MIC_starved_ = 0.04 µM [135]	-
**6**	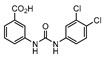	CysM	-	3-log [143]
**7**	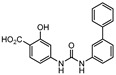	CysM	-	3-log [143]
**8**	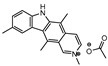	APSR	MBC_nonreplicating_ = 1.5 µM [144]	-
**9**	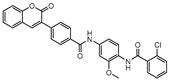	DevR	-	4-log [162]
**10**	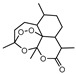	DevRST	EC_50_ = 1.2 µM [163]	-
**11**	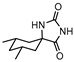	DevRST	EC_50_ = 12.4 µM [163]	-
**12**	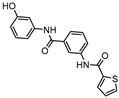	DevRST	EC_50_ =2.7 µM [163]	-
**13**	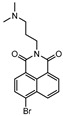	DevRST	EC_50_ = 2.8 µM [164]	-
**14**	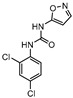	DevRST	EC_50_ = 6.9 µM [164]	-
**15**	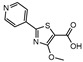	LAT	IC_50_ LAT = 1.22 µM [169]	2.8-log [169]
**16**	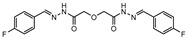	LAT	IC_50_ LAT = 0.81 µM [170]	2-log [170]
**17**	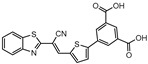	LAT	IC_50_ LAT = 2.62 µM [171]	2.9-log [171]
**18**	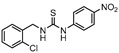	InhA	-	2.6-log [175]
**19**	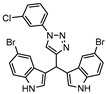	DprE1	IC_50 dormant_ = 0.12 µg mL^−1^ [181]	-
**20**	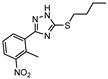	CYP121	IC_50 dormant/active_ < 0.03 µg mL^−1^ [185]	-
**21**	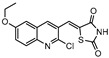	Zmp1	IC_50 active_ = 2.3 µg mL^−1^ [189] IC_50 dormant_ = 1.9 µg mL^−1^ [189]	-
